# Supplementary material for: Causal interplay between lactose intolerance and gut microbiota: a combined bidirectional Mendelian randomization and in vivo validation study
Source: Front Nutr. 2026 Jun 1;13:1803337. doi: 10.3389/fnut.2026.1803337 (PMC13265576; doi:10.3389/fnut.2026.1803337)
Supplement: Supplementary file 1 [file Data_Sheet_1.zip › supplementary materials/Forward/leave one out cross validation/ebi-a-GCST90027536.finngen_R12_E4_LACTONAS.pdf]

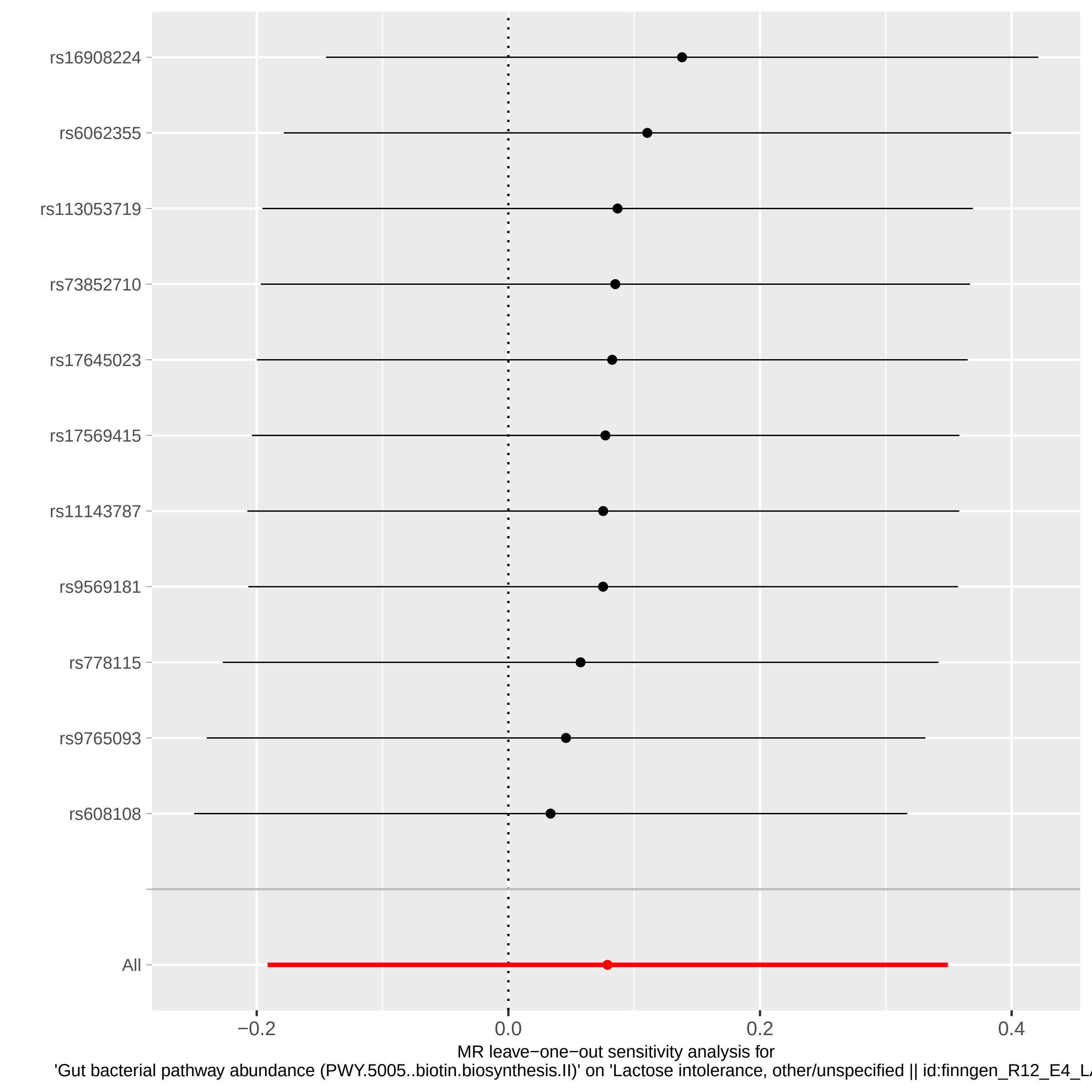
rs16908224
rs6062355
rs113053719
rs73852710
rs17645023
rs17569415
rs11143787
rs9569181
rs778115
rs9765093
rs608108
All
−0.2
0.0
0.2
0.4
MR leave−one−out sensitivity analysis for
'Gut bacterial pathway abundance (PWY.5005..biotin.biosynthesis.II)' on 'Lactose intolerance, other/unspecified || id:finngen_R12_E4_L
